# Physical Punishment in Childhood and Adolescence: Related Factors and Outcomes

**DOI:** 10.1007/s40653-025-00725-x

**Published:** 2025-06-23

**Authors:** Klara Svalin, Marie Torstensson Levander, Sten Levander

**Affiliations:** Department of Criminology, Malmö University, Malmö, Sweden

**Keywords:** Physical punishment, Family bonds, Peer relations, Sex differences, Self-regulation, Antisocial behaviour, Criminality

## Abstract

**Background:**

Physical punishment (PP) of children is banned in > 50 countries, motivated by ethical arguments as well as empirically ascertained negative effects in adulthood. There is ample social pressure against PP in Sweden.

**Aims:**

How common is a history of PP among randomly selected Malmö adolescents, is PP explained by certain social background factors and/or individual characteristics, and how do physically punished individuals differ with respect to antisocial outcomes at age 18.

**Method:**

Participants (*N* = 518, a 20% sample of children born in Malmö 1995) responded to a comprehensive self-report questionnaire in three waves, age 15, 16 and 19.

**Results:**

PP was reported by appr. 20% of the participants. Several differences were observed between participants who experienced PP and those who did not. These differences can be summarized as follows: parents’ country of birth, the importance of religion, conflicts with parents, poor bonding with family, exposure to antisocial peers, perceived victimization by peers and teachers, victimization from crime, and difficulties with certain aspects of self-regulation.

**Conclusion:**

In spite of being illegal, PP is relatively common. There were some significant associations suggesting differences between those exposed to PP and the others, but no serious effects at age 19, except violent behaviour for boys.

**Supplementary Information:**

The online version contains supplementary material available at 10.1007/s40653-025-00725-x.

## Introduction

During the Enlightenment, Rosseau introduced a new perspective on the upbringing of children, advising against physical punishment. Almost 200 years later, this advice based in the humanities was backed up by a natural science approach – physical punishment as well as parental abuse/battery and neglect is empirically harmful (Larzelere & Kuhn, 2005; APA, 2019; Glicksman, 2019). In her classic text, Widom (1989) introduced the concept of *cycle of violence*. It summed up decades of discussions concerning physical punishment of children. Already, 10 years earlier, such punishment was banned by law in Sweden. Since then, many studies have reported that physical punishment of a child predicts long-term negative consequences in adulthood (Reavis et al., 2013). However, there are also studies that do not confirm such a relationship, suggesting that the causal mechanisms are complex (Myers et al., 2018). These authors made a re-analysis of Widom’s original data set and to some extent replicated her conclusions. However, robustness checks failed: childhood neglect rather than physical punishment emerged as a robust predictor of adult violence. In a previous study (Ivert et al., 2011), we noted that boys appeared to be more vulnerable in that respect.

There are many suggested generative mechanisms linking violence to psychological mechanisms (Allen et al., 2018; Heleniak & McLaughlin, 2020; Sturmey, 2022). Mediation may be similar to that of a post-traumatic stress disorder (PTSD) – which requires exposure to a serious negative life event. PTSD in late adolescence is associated with a 13-fold increase in self-reported violent crimes (Coker et al., 2014). Suggestions for generative mechanisms can be deduced from the list of PTSD symptoms according to DSM-5 (North et al., 2016; Svingen, 2023). Attention problems, a lower threshold for aggressive acting out, suspiciousness, and social distancing are all associated with increased violence in many studies (Allen et al., 2018).

Another set of theoretical concepts was tested in a large-scale study based on data from 26 countries, including more than 61,000 participants (Manzoni & Schwarzenegger, 2019). The authors examined whether five different variables (*family bonds*,* school bonds*,* self-control*,* moral values*, and *delinquent peers*) mediated the relationship between parental abuse and violent delinquency. All variables were significant predictors of violence in cases subjected to parental abuse, with poor self-regulation and delinquent peers being strongest.

Furthermore, some studies suggest that a high trait level of Negative affect (depression/anxiety), which in turn may have been induced by physical punishment, is associated with negative effects on social adaptation in adulthood (Grove et al., 2016). This study focused on psychotic disorders but included controls for whom the same findings were obtained. Manzoni and Schwarzenegger (2019) found that exposure to parental maltreatment was associated with a lower level of morality. Some studies report that physical punishment interferes with a child’s cognitive development (e.g., Salhi et al., 2020). Heilmann et al. (2021) did a meta-analysis of 69 prospective longitudinal studies of physical punishment. One main finding was that externalising behaviour and diagnoses with such problems had the highest level of shared variance with physical punishment. Another finding was the strong linear dose-response effect of physical punishment on a range of negative outcome variables, suggesting that physical punishment carries causal power.

### Judicial Context

In 1979, physical punishment was banned in Sweden, the first country to do so (Kvist et al., 2020). A few years later, Gelles and Edfelt (1986) examined and compared the prevalence of violence towards children in Sweden and the United States, based on the large discrepancy in social attitudes in relation to violence (e.g., differences in laws, varying availability of weapons, and the extent of violence shown on television). Just over 50% of the caregivers in the Swedish sample reported that they had ever used violence (hit) their children. The corresponding numbers in the US sample was just over 70% (this sample was asked whether they had slapped or spanked their children). Around 63% in the Swedish sample reported that they had pushed, grabbed, or shoved children, as compared to 46% in the US. The overall conclusion was that the differences in the use of violence among caregivers in Sweden and the US were smaller than expected when considering differences in social attitudes in relation to violence.

In a more recent study, Kvist et al. (2020) evaluated the prevalence of caregivers’ physical violence among children ages 15 to 17 in 2008, 2011, 2014, and 2017 in Sweden. The data set was based on children’s self-reports. There was a decline in violence exposure: 19% in 2008 to 13% in 2017. The prevalence of repeat violence also declined significantly during the period, but not to the same extent, probably reflecting a change in social attitudes to violence against children.

The ban of physical punishment currently affects > 50 countries (End Violence Against Children, *a UNICEF-hosted fund*, 2021). The ban is limited in, for instance, the UK where it is banned in school but not at home. Sweden has welcomed more immigrants per inhabitant than almost any other country. Many have origins in non-Western cultures with different disciplination traditions. Malmö, where the present study was conducted, is a city with particularly many first and second-generation immigrants. In line with the findings of Naudin et al. (2023), we expected differences in the use of PP among families with different ethnic backgrounds.

### Aim and Research Questions

The aim of the study is to investigate how common a history of physical punishment is among randomly selected adolescents in a Swedish city. Furthermore, we explored whether the use of physical punishment was associated with prior and contemporary factors like sex, family, school, peers and individual characteristics. Furthermore, we examined whether physically punished individuals differ with respect to crime and antisocial outcomes at age 18.

## Method

### Material, Design, and Procedure

The current study is based on data from the Swedish longitudinal study The Malmö Individual and Neigbourhood Development Study (MINDS; Chrysoulakis et al., 2025; Engström, 2018; Ivert et al., 2018; Nilsson et al., 2021). The overall aim of the MINDS study is to contribute to a better understanding of the causes and prevention of young people´s involvement in crime. The sample consists of approximately 20% of all persons born in 1995 and living in Malmö 2007 (*N* = 525). MINDS participants were selected as a random sample of the cohort. MINDS (Dnr. 201/2007) was approved by the Swedish Regional Ethical Review Board in Lund. The number of participants is presented in Table 1. About 50% of the sample was girls. With respect to ethnicity, 63% of the participants had Swedish- or Nordic-born parents. At least one parent had another European background in 14% of the cases, 10% had a Middle Eastern, and 7% an Asian background. The remaining participants (10%) had parents with other ethnic backgrounds. The participants filled in a comprehensive self-report questionnaire at ages 15, 16, and 19 (Wave 2, 3 and 4). With the exception of individual traits and criminality, all data refer to Wave 2 and 3.

**Table 1:** Number of participants in the waves

		2 & 3	2 & 4	2 & 3 & 4
Wave 2	514	482	393	386
		3 & 4		
Wave 3	517	403		
Wave 4	412			

### Data and Variables

#### Physical Punishment

Physical punishment (PP) was reported using four items. In wave 2 and wave 3, the participants answered the question: *‘During the past school year*,* have your parents slapped you or done something similar because you behaved badly*,* and how many times?’* In wave 2, they also answered whether this had ever happened. They were also asked if this had occurred during the past school year. From this, we created two separate variables measuring prevalence and frequency.

#### Family, School, and Peers

We use three groups of variables to measure aspects of family, school, and peers reported by the participants in waves 2 and 3. The first group consists of the parents’ (mothers) country of birth (*Sweden/ Europe/ a country outside Europe*), and the importance of religion for the family and the participant.

The second group includes measures of Family bonds which is a combined scale of parental *attachment and monitoring (*Cronbach’s alpha = 0.80). Examples of items are: ‘*How often do you usually have dinner with your parents?’*,* ‘Do you talk to your parents about how you are doing in school?’*,* ‘How well do you get along with your friends?’*, and *‘When you are out by yourself or with friends*,* do your parents usually know where you are*,* with whom*,* and what you are doing?’.* This group also contains Conflicts with parents measured by the items: ‘*How often do you argue with or get angry at your parents?’*, ‘*Would your parents (step-parents) be very angry with you if you misbehaved (for example*,* if you talk back to them or did not do what they told you to)?’* and ‘*Would your parents (stepparents) punish you if you misbehaved?’*

School bonds was defined as school attachment by the item: *‘Do you like school?’* and commitment to school by items: ‘*How many hours per day do you usually spend on homework for school?’* and *‘If you could leave school tomorrow*,* what would you do?’*

The third group indicates Exposure to violent peers and was based on the items: *‘Do your closest friend/s fight*,* or get into fights?’* and ‘*Do your closest friend/s destroy things that do not belong to them (e.g.*,* smashing windows*,* scribbling*,* or scratching the paint on cars)?’*

Victimisation is defined as feeling unsafe and/or being bullied, and/or treated unfairly by teachers, and/or being victimised to crime (hit or kicked, exposed to stealing and robbery).

#### Self-regulation, Aggression, and Morality

Self-regulation was assessed using 20 self-report items in all waves. Of these, eight refer to a short version of the Grasmick scale (Wikström et al., 2012), and the other items reflect morality and additional self-regulation items including Aggression problems.

#### Personality (SDQ Traits and NA State)

The Strength and Difficulties Trait Questionnaire (SDQ, 25 items) and five items reflecting state negative affect (NA) were filled in at each wave. We wanted to be able to compare the three data waves – hence, the factor structure of the SDQ instrument had to be forced into one dimensional structure valid for the three waves. We ended up with four dimensions: ADHD-type signs/symptoms, Negative affect, Social desirability, and Inter-personal problems. The single item/total correlation was > 0.29 and the r_iccc_ >0.19 for these subscales. The four dimensions were approximately orthogonal. The State negative items scales were only moderately correlated with the corresponding wave/trait ones, suggesting that they did reflect ‘state’.

#### Criminality, Antisocial Behaviour, and Drug Use

Criminality at age 15 (wave 2) and 16 (wave 3) was based on four self-report items of non-violent crimes (shoplifting, theft, car theft, burglary), and four items of violent crimes (assault, vandalism, robbery, arson) committed during the last year, forming a set of *crime indices*. Three pairs of items reflected age at first use and frequency of cigarettes, alcohol (becoming drunk), and cannabis, and current use (waves 2 and 3), forming a set of *abuse indices*. Three items referred to norm-breaking behaviours such as having run away from home and truancy. These indices were summed to indices of less serious kinds of *antisociality*.

### Missing Values

A small number of scattered missing values (no more than 4% for any variable) were imputed, either as the most common value or based on a linear regression for relevant items. All items were scanned with respect to mis-entering or obvious inconsistencies and were corrected.

### Analytical Strategy

The data set includes variables which can be viewed as independent as well as dependent. At the core are variables reflecting physical punishment (PP), in some analyses dependent (does parents’ country of birth affect PP?), and in others independent (does PP increase abuse in late teens?). The time aspect governs if a measure is independent or dependent (parents’ country of birth precedes PP). Before that stage, conceptually similar items were analysed separately with the aim to form relevant and homogenous summary variables, e.g., an ADHD-type, poor self-regulation index and a summary index over waves of more serious criminality. Armed with a comprehensive set of summary variables, we tested associations between variables two at a time, using simple statistical methods (t-test, chi-square test, Kendall’s Tau) and governed by the research questions. Whenever possible, we tried to estimate the *clinical significance* (effect size) of the association. Sex is an independent variable in all analyses. However, since the girls are underrepresented when it comes to crime in Wave 4 we do not conduct separate analyses for boys and girls. Finally, a binary logistic regression analysis was conducted with PP as dependent variable and variables (wave 2 and 3) that reached significance in relation to PP in the bivariate analyses, as independent variables.

## Results

In the first section, self-reports of physical punishment among the participants are presented and analysed in relation to the separate groups of variables as referred to in the previous section.

### Physical Punishment (PP)

Twenty per cent of waves 2 and 3 participants checked at least one of the five variables pertaining to physical punishment. PP before age 15 (pre-adolescence) was reported by 18%. It was repeated over at least two time periods in 4.4% and three time periods in 2.2%. There were no sex differences.

### Family Aspects, School Bonds, and Peer Influence

Parents’ country of birth was significantly related to PP. Children with mothers born in countries other than Sweden had been physically punished to a higher extent than children with parents born in Sweden (chi-square 14.10, df = 1, *p* < 0.001). The importance of religion for the family was moderately and positively associated with PP (tau = 0.16, *p* < 0.001); and slightly less associated with the importance of religion for the participant (tau = 0.15, *p* < 0.001).

Strong family bonds reduced PP for boys (t = 3.71, *p* < 0.001, d = 0.56), but not for girls. Conflicts with parents, on the other hand, were strongly associated with PP for girls (t = 3.94, *p* < 0.001, d = 0.63), but less so for boys (t = 1.83, *p* = 0.04, d = 0.27). There were no significant effects of PP in relation to school bonds for either boys or girls.

With respect to contacts with antisocial peers, two items from wave 2 and two items from wave 3 were combined (r_iccc_= 0.41) and analysed in relation to PP. There was a significant effect of PP for both girls (t = 1.64, *p* = 0.05, effect size d = 0.26 i.e., small), and boys (t = 2.10, *p* = 0.02, effect size d = 0.32).

### Victimisation

Three subscales for waves 2 and 3 were computed, and all were strongly negatively skewed. All scales were positively associated with PP for girls, Unsafe and/or bullied (t = 2.91, *p* = 0.002, d = 0.45), Victimised to crime (t = 2.33, *p* = 0.01, d = 0.47), and Unfair teachers (t = 2.77, *p* = 0.003, d = 0.40). For boys, only Unfair teachers was associated with PP, (t = 4.62, *p* < 0.001, d = 0.78).

### Self-regulation, Aggression and Morality

We used the self-regulation scale extracted in a previous study of the MINDS cohort (Levander & Torstensson Levander, 2024, see Appendix 1.) reflecting aggression control, impulsiveness/attention, morality and executive functions. The Impulsiveness/attention and Aggression control factors for waves 2 and 3 were analysed separately for sex and yielded significant differences. Impulsiveness/attention problems were more extensive for boys exposed to PP (t = 2.68 and 2.70, *p* < 0.01, d = 0.44 and 0.44). The patterns for girls were quite similar (t = 2.44 and 2.63, *p* < 0.05, d = 0.41 and 0.44). For boys, the Aggression indices were higher for those exposed to PP (t = 3.04, *p* < 0.01, effect size d = 0.5). For girls the difference was moderately significant (t = 1.40, *p* = 0.08 d = 0.24). The executive functions and morality factors did not differ significantly.

Regarding the SDQ-scale, four dimensions were identified to summarise the three waves of SDQ items. The dimensions were characterized as ADHD-type problems, Negative affect, Social desirability, and Interpersonal problems. Wave scores were summarised to one, single scale. ADHD scores were higher for physically punished adolescents, including boys as well as girls (t = 2.58 for boys, 2.54 for girls, both *p* < 0.05, effect size small). The other dimensions were not associated with PP.

### Criminality, Antisocial Behaviour, and Drug Use

Overall, there were no significant differences between children exposed to PP and the others with respect to criminality and antisocial behaviour in waves 2 and 3, respectively.

For wave 4, we tested the relation between PP, theft and violent crimes, respectively (versatility scale), and antisocial behaviour (running away from home and truancy). Theft showed no significant relation to PP, violent crimes were significantly related to PP (chi-square 17.617, df = 12, *p* < 0.001). Also, antisocial behaviour was significantly related to PP (t-test 3.558, *p* < 0.001).

Regarding drug use, only one comparison was statistically significant (*p* < 0.01): girls exposed to physical punishment (PP) used cannabis more frequently than girls without PP exposure. The effect size (Cohen’s d) was around 0.5. Therefore, these findings may be spurious. Overall, exposure to PP is not explained by the use of psychoactive substances, nor does exposure to PP appear to lead to the use of psychoactive substances.

Finally, we conducted a binary logistic regression analysis with PP as dependent variable (see Table 2). Four variables reached significance (*p* < 0.05): Family bonds, Conflicts with parents, Parents’ country of birth and Unfair teachers.

**Table 2:** Binary logistic regression. Dependent variable = Physical punishment

Variables	Exp(B)	*P*-value	95% Confidence Interval for Exp(B)	
Parents’ country of birth (0 vs. 1)*	2.92	0.007	1.339	6.347
Parents’ country of birth (0 vs. 2)*	2.15	0.026	1.095	4.205
Family bonds	0.93	0.030	0.878	0.993
Conflict with parents	0.85	0.016	0.739	0.970
Exposure to violent peers	0.95	0.534	0.799	1.123
Unsafe and/or bullied	1.07	0.234	0.958	1.190
Unfair teachers	1.16	0.012	1.033	1.305
Victimised to crime	1.00	0.923	0.902	1.098
Aggression	0.99	0.836	0.901	1.088
ADHD-type signs/symptoms	1.04	0.329	0.957	1.139
Antisocial behaviour	1.08	0.151	0.972	1.202

*0 = Sweden, 1 = Europe, 2 = Country outside Europe

Nagelkerke R Square: 0.20

## Discussion

Physical punishment (PP) of children and youth, which is considered a crime under Swedish law, was used in 20% of the families, with repeated instances occurring in 10%. Several differences were observed between participants who experienced PP and those who did not. These differences can be summarized as follows: parents’ country of birth, the importance of religion, conflicts with parents, poor bonding with family, exposure to antisocial peers, perceived victimization by peers and teachers, victimization from crime, and difficulties with certain aspects of self-regulation.

Several personality-related factors are strongly linked to physical punishment. A reasonable assumption is that believing teachers are unfair, having parents with a punitive parenting style, and having weak bonds with parents may indicate that the young person behaves in a way that provoke the parents. The overrepresentation of children with parents from foreign backgrounds can likely be explained by the fact that their countries of origin may not have laws regulating corporal punishment, and such behavior is often considered acceptable in those cultures (cf. Naudin et al., 2023). However, it is important to remember that even among children of Swedish origin, about 18% report having been subjected to violence by their parents.

An important finding is the significant correlation between physical punishment among boys (only a small number of girls reported violent crimes) and violent crimes at age 19. However, we cannot draw definitive conclusions about the causal relationship between these variables, nor can we say whether violence serves as a marker for underlying factors, such as personality traits (particularly aggression), that increase the risk of committing such crimes.

Can we draw any conclusions about the negative consequences of physical punishment for a child or youth’s life course? Overall, the negative effects observed at age 19 appear to be small, likely because the physical punishment was mild and the parents can generally be assumed to be well-adjusted. However, when it comes to negative life events that lead to PTSD, the situation is quite different—here, the consequences may be substantial (Mossige & Huang, 2017; Beaudry et al., 2021).

Is it necessary to demonstrate that physical punishment (PP) has negative effects? Not necessarily. The rationale for minimizing PP is primarily ethical or a question of moral values.

## Conclusions

Some implications can be drawn from the present study. Among youths exposed to PP, externalising problems are often accompanied by executive functioning deficits and negative perceptions of others – factors that can contribute to interpersonal conflicts, which these individuals may struggle to manage. These difficulties warrant support and intervention. Strengthening parent–child bonds—which are known to be protective—should be a key focus of such efforts. Supportive approaches and treatment are more effective than punitive sanctions, which are not only ineffective but may exacerbate conflict (Glicksman, 2019).

Furthermore, the current dataset has potential for deeper analysis using the comprehensive criminological framework of Situational Action Theory (Wikström et al., 2024), especially concerning the development of aggression and violent behaviour (Wiggers & Paas, 2022).

## Electronic Supplementary Material

Below is the link to the electronic supplementary material.

Supplementary Material 1

## Data Availability

Not applicable.
