# Supplementary material for: Physical Punishment in Childhood and Adolescence: Related Factors and Outcomes
Source: J Child Adolesc Trauma. 2025 Jun 23;18(4):841–7. doi: 10.1007/s40653-025-00725-x (PMC12831742; doi:10.1007/s40653-025-00725-x)
Supplement: Supplementary file 1 — Supplementary Material 1 [file 40653_2025_725_MOESM1_ESM.docx]

| Question | origin | factor | Extr. |
| --- | --- | --- | --- |
| When I am really angry other people better stay away from me. | G | AGGR | 0.84 |
| I often act on the spur of the moment without stopping to think. | G | ADHD | 0.55 |
| I always react with poor conscience when I do something wrong | M | MORAL | 0.67 |
| I tend to become irritated or crossed on other people. | S | - | 0.50 |
| I sometimes find it exciting to do things that may be dangerous | G | ADHD | 0.78 |
| I do not devote much thought and effort preparing for the future. | G | EXEC | 0.73 |
| Sometimes I will take a risk just for the fun of it | G | ADHD | 0.78 |
| If I do something that upsets people, it is their problem, not mine. | S | ADHD | 0.57 |
| I often try to avoid things that I know will be difficult. | G | EXEC | 0.40 |
| I do not care much if other people think that I act wrongly. | M | MORAL | 0.54 |
| I never think about what will happen to me in the future. | G | EXEC | 0.71 |
| I always try to avoid to hurt and harm people. | M | MORAL | 0.60 |
| I get bored easily. | S | ADHD | 0.43 |
| I often feel stupid when I do something that is wrong | M | MORAL | 0.70 |
| I lose my temper pretty easily | G | AGGR | 0.74 |
| I always get a bad conscience when I am late paying back money to friend | M | MORAL | 0.43 |
| When I get angry I have difficulties to control what I do. | S | AGGR | 0.87 |
| If I feel tempted to do something which I should not do, I will often do it | S | ADHD | 0.73 |
| If I feel tempted to do something which I should not do, I never think about problems that might happen – be detected or be caught | S | - | 0.50 |
| When I get angry I never think about the consequences of what I do | S | AGGR | 0.79 |

Appendix 1. The 20 Grasmick self-control/morality (GSC/M) items.^[[1]](#footnote-1)^

Origin: G(rasmick), M(oral), S(elfcontrol). Facor 1: Aggression control. Factor 2: ADHD problems. Factor 3: Morality. Factor 4: Executive. - not used. Extraction coefficients for the factor is provided.

1. Levander & Torstensson Levander, 2024 [↑](#footnote-ref-1)
